# Supplementary material for: Mechanism for Stabilizing an Amorphous Drug Using Amino Acids within Co-Amorphous Blends
Source: Pharmaceutics. 2023 Jan 19;15(2):337. doi: 10.3390/pharmaceutics15020337 (PMC9964172; doi:10.3390/pharmaceutics15020337)
Supplement: Supplementary file 1 [file pharmaceutics-15-00337-s001.zip › pharmaceutics-2111669-supplementary.pdf]

Supplementary Material

Mechanism for stabilizing an amorphous drug by amino acids within co-amorphous blends

Yannick Guinet, Laurent Paccou and Alain Hédoux

Alain.hedoux@univ-lille.fr

Univ. Lille, CNRS, INRAE, Centrale Lille, UMR 8207 - UMET - Unité Matériaux et Transformations, F-59000 Lille, France

https://umet.univ-lille.fr

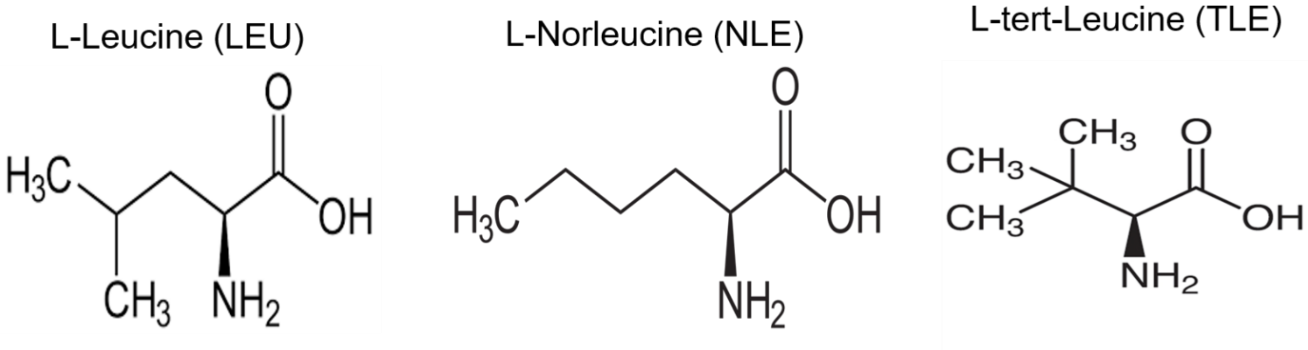

Figure S1. Chemical structure of amino acids

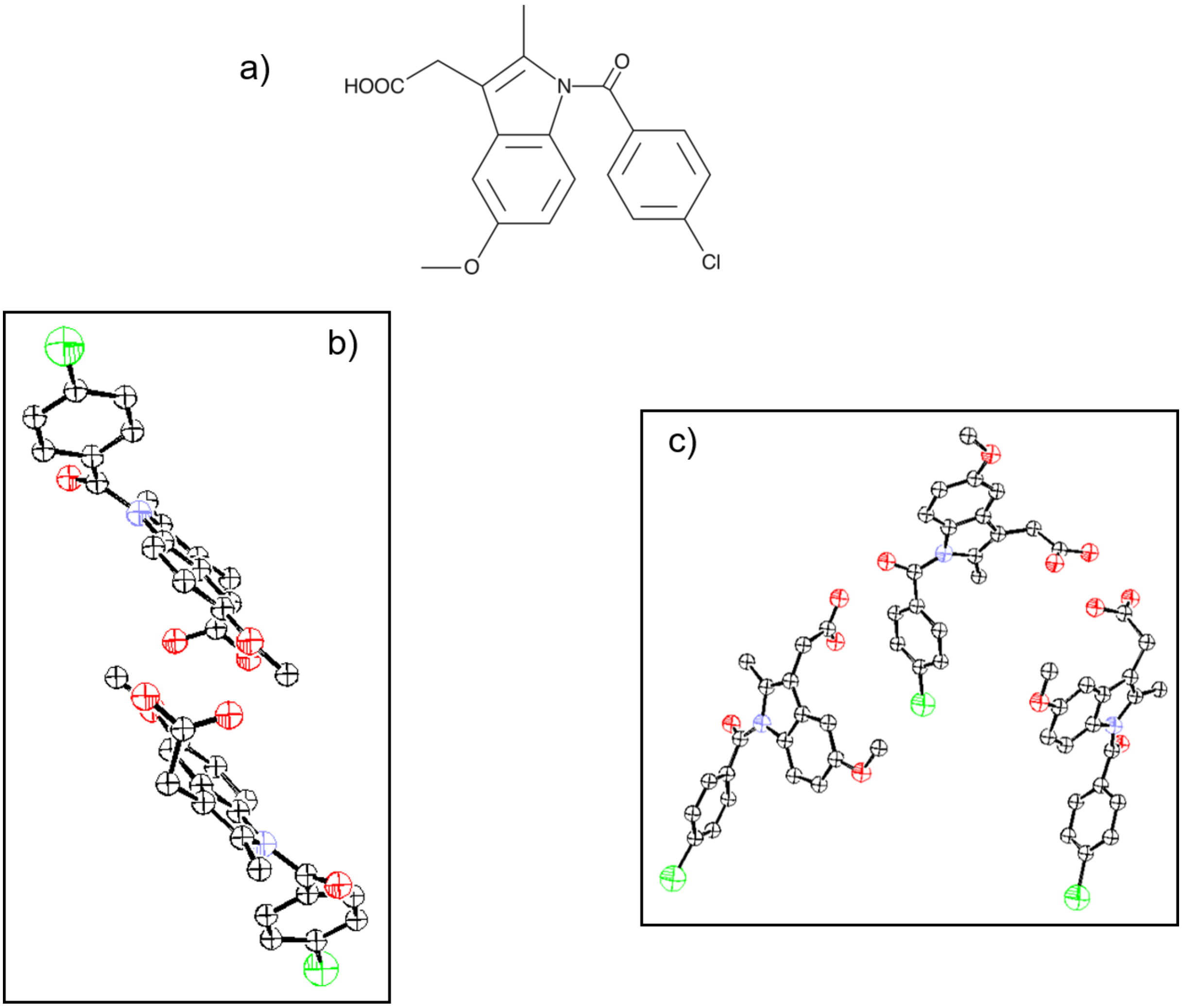

Figure S2. Structural description of indomethacin; a) chemical structure; b) molecular packing (dimers) in γ phase; c) molecular packing (trimers) in α phase.

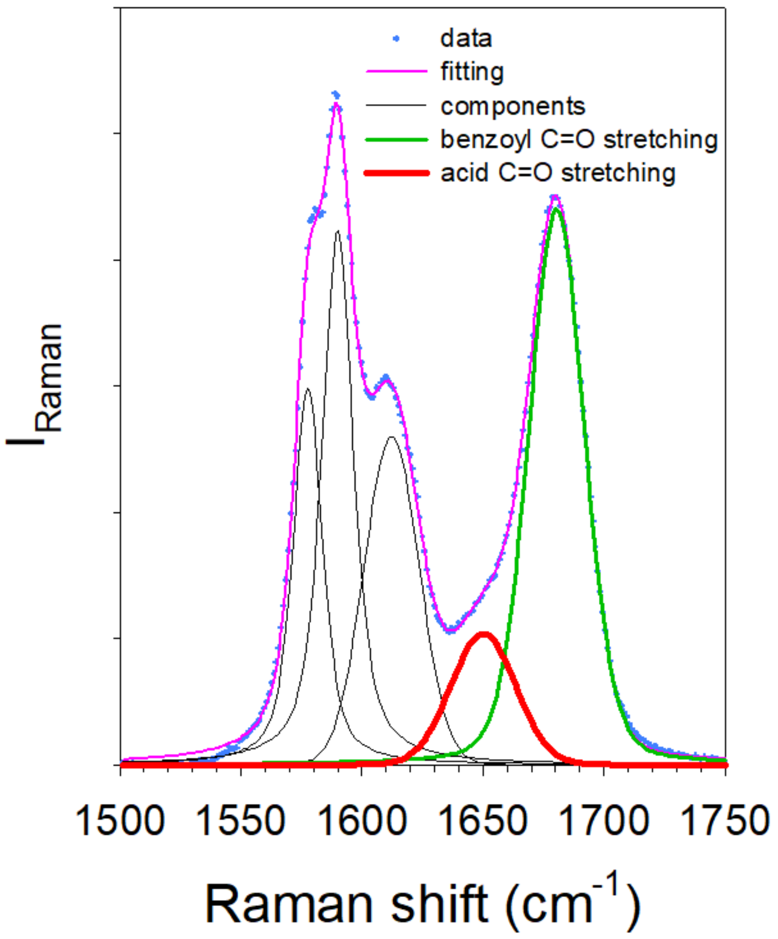

Figure S3. Description of the fitting procedure of the C=O stretching spectrum in indomethacin; the Raman band plotted in thick green which exhibits the signature of H-bonding molecular associations was analyzed in the present study

Table S1: assignment of Raman bands in the 1550 – 1750 cm-1 region of the spectrum of indomethacin

Raman band (cm^{-1})	form	assignment
1588	γ	
1589	amorphous, δ	ring vibration of indole
1591	α	
1611	amorphous,α	
1614	δ	C = C stretching
1618	γ	
1648	**amorphous**, α	H bonded acid C = O stretching
1673	δ	H bonded benzoyl C = O stretching
1678	α	
1679	**amorphous**	(Non-H bonded + H bonded) benzoyl C = O stretching
1685	δ	
1688	α	non-H bonded benzoyl C = O stretching
1697	γ	benzoyl C = O strretching

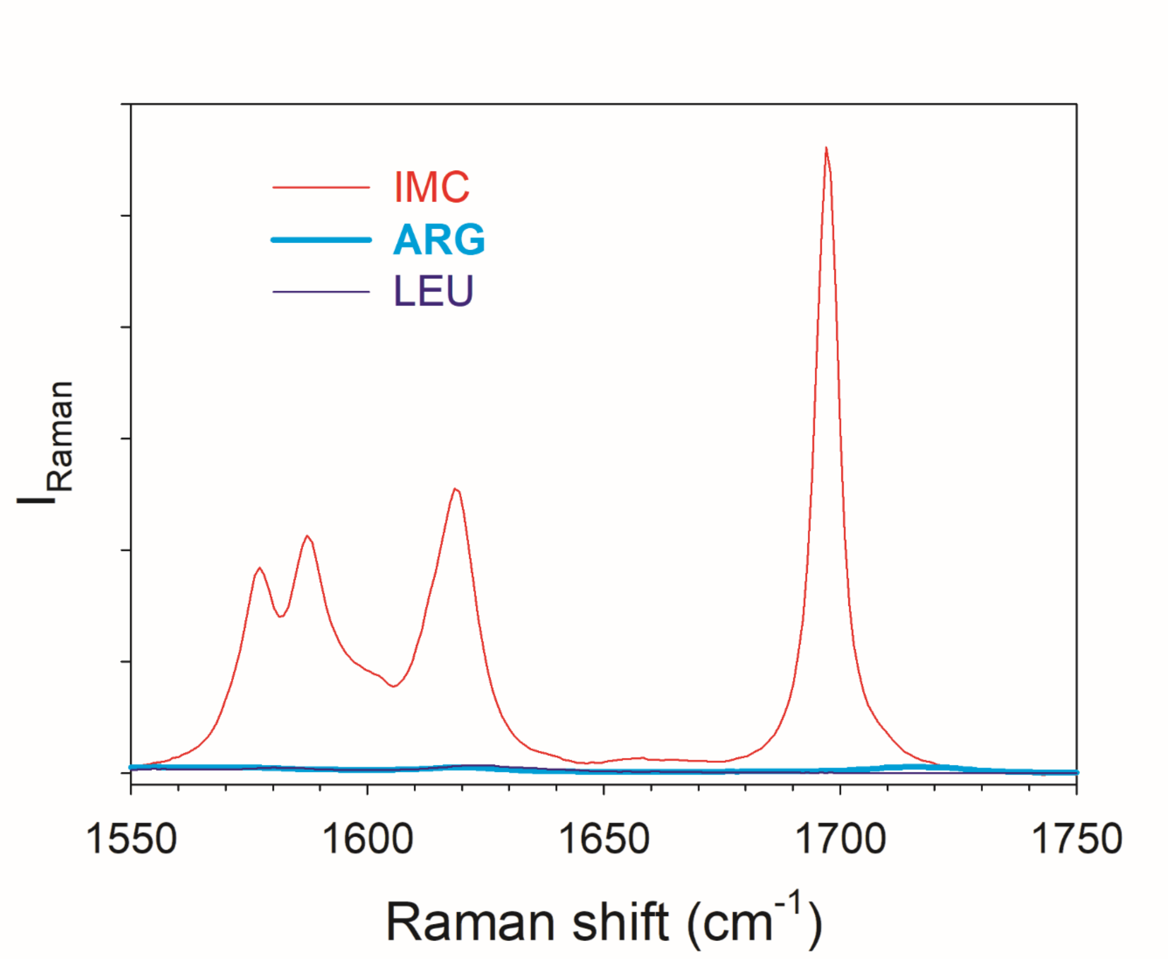

Figure S4: comparison between Raman signals scattered by pure compounds in the crystalline state, indomethacin (IMC, gamma phase) and leucine (LEU), arginine (ARG) in the mid-frequency region. Spectra were collected in the same experimental conditions, including acquisition time.

Microcalorimetry measurements were carried out on a very sensitive microcalorimeter microDSC III (Setaram Instrumentation, Caluire, France). Sample mass of 400 mg was used for IMC-ARG mixture blend. The calibration was performed using water and naphthalene (T_m ~ 80 °C) Heating run was performed with a scanning rate of 1 °C/min (same scanning rate used for low-frequency Raman spectroscopy). It is noticeable that the glass transition is spreading out over about 15 degrees, between 60 and 76 °C.
Raman experiments were performed on samples (~ 50 mg) are placed in Pyrex spheres hermetically sealed. The spheres were placed within a regulated nitrogen flux, necessarily inducing a temperature gradient between the set point temperature (thermocouple sensor located in the nose of the device) and the temperature of the sample inside the Pyrex sphere. The two types of heat transfer can partly explain the difference the two determinations of T_g.

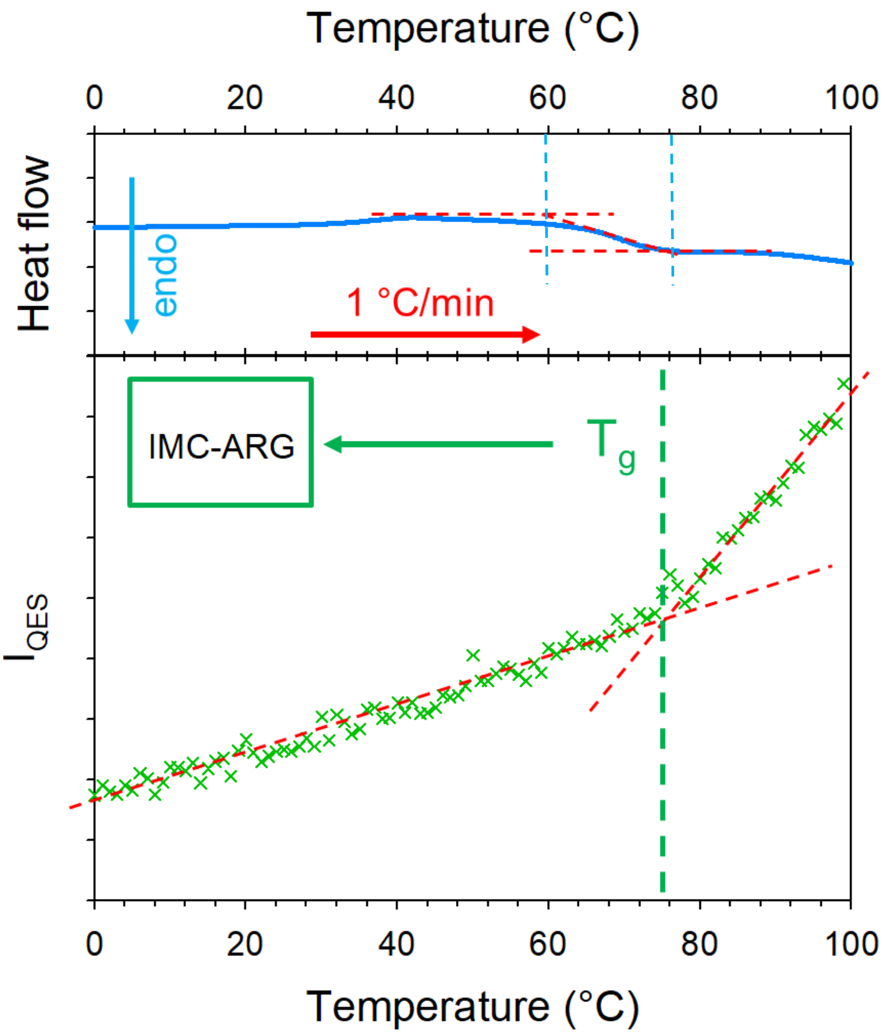

Figure S5: comparison between DSC curve and temperature dependence of quasielastic intensity; both calorimetric and Raman data were collected with the same scanning rate (1 °C/min) and provide approximatively the same value of T_g. Dashed lines are guides for the eyes.

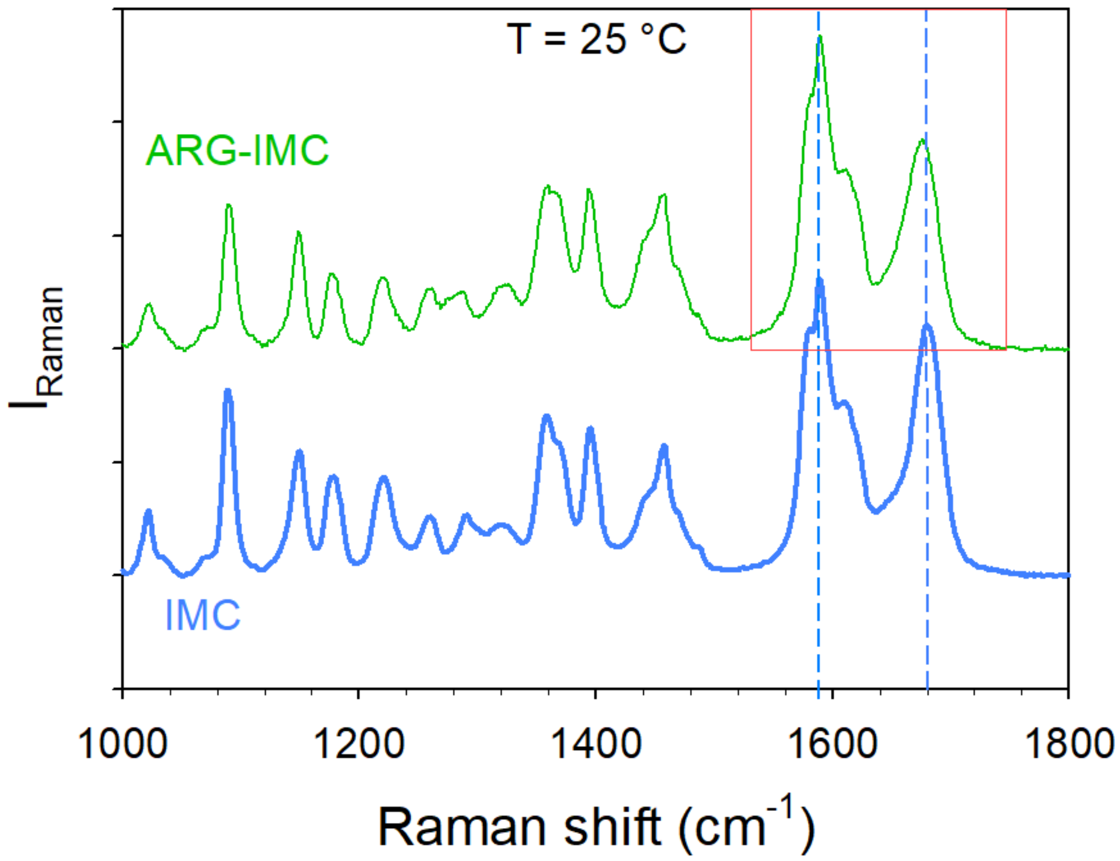

Figure S6: comparison between mid-frequency spectra of indomethacin (IMC) and co-amorphous indomethacin-arginine (IMC-ARG). This comparison indicates the absence of changes in the molecular conformation of IMC within the IMC-ARG co-amorphous blend. The red frame corresponds to the spectral region selected for analyzing H-bonding in co-amorphous blends from the fitting procedure plotted below which can be compared with that of IMC plotted in Figure S3. The blue vertical dashed lines show that only the high-frequency bands (> 1650 cm^{-1}) in the co-amorphous mixture are shifted towards the low-frequencies.

Fitting procedure used to analyze the temperature dependence of the 1680 cm^{-1} band in IMC-ARG co-amorphous blends

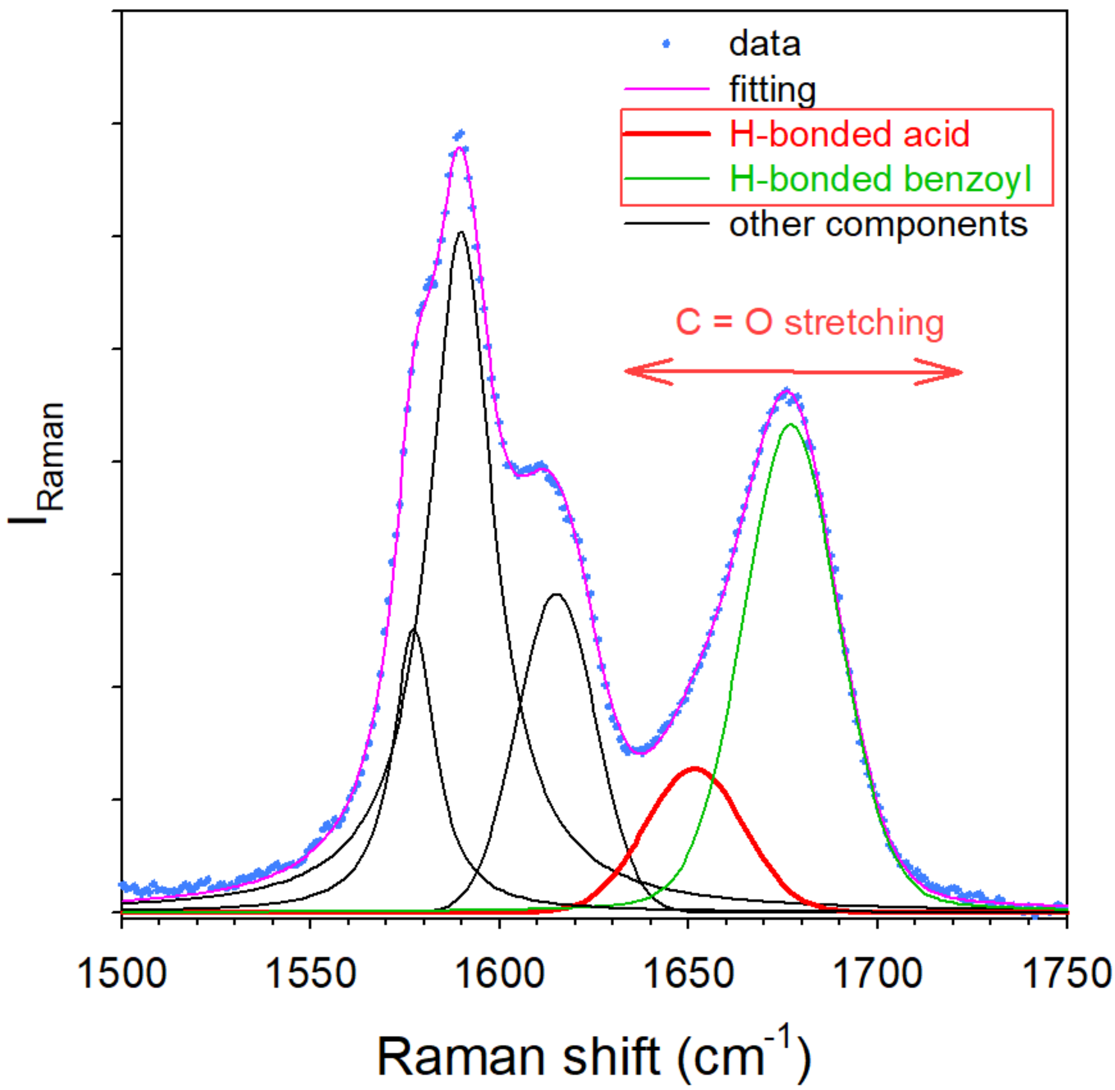

Figure S7: fitting procedure of the IMC-ARG spectrum at room temperature which can be compared with that of IMC in Figure S3. It is observed that the C=O stretching region is composed of two bands as in pure IMC, and only a shift of the C=O stretching bands can be detected between IMC and IMC-ARG.

It is noticeable that this fitting procedure shows that the 1500 – 1750 cm^{-1} region in IMC-ARG is composed of Raman bands existing in pure amorphous IMC (see Figure S3).
